# Chloral Hydrate to Study Auditory Brainstem Response

**DOI:** 10.1590/S1808-86942010000400005

**Published:** 2015-10-19

**Authors:** Mariana Lopes Fávero, Fabiana Amaral Sanches Ponce, Marcio Ricardo Barros Pio, Alfredo Tabith, Fernando Leite Carvalho e Silva

**Affiliations:** a PhD in Sciences - Otorhinolaryngology Department - FMUSP, ENT - DERDIC/PUCSP and HSPM; b Otorhinolaryngologist - ABORL-CCF, and DERDIC/PUCSP; c Otorhinolaryngologist - ABORL-CCF; d MSc in Human communicaton Disorders - PUC/SP, Phoniatrist - Head Clinician - DERDIC/PUCSP; e Phoniatrist - PUC/SP, and DERDIC/PUCSP. ENT and Phoniatry Department - DERDIC/PUCSP

**Keywords:** pediatrics, evoked potentials, deep sedation

## Abstract

Chloral Hydrate (CH) is a sedative and hypnotic drug used in pediatric procedures owing to the low depressive effect it has on the respiratory and cardiac systems.

**Aim:**

To assess the efficacy of the drug in performing ABR and to systematize its use.

**Materials and Methods:**

A prospective cross-sectional study with 41 children without history of heart or lung disease. The initial dose of CH at 10% was 50mg/Kg, with a boost dose of 6mg/Kg administered 30 minutes later in cases in which there was no sedation. Drug effectiveness was established by sleep induction by 1 hour after the administration of the initial dose. Sleep occurrence was correlated with doses (50mg or 56mg/Kg), age, weight and gender.

**Results:**

All the 41 children who participated in the study took 50mg/kgof the agent and 23 of them slept within 30 minutes, 2 had respiratory complications, 16 had the 6mg/Kg boost dose and 13 fell asleep after 30 minutes. The 56 mg/kg dose presented a statistically significant effect on sleep induction (p<0.05) when compared to the 50mg/kg dose.

**Conclusion:**

CH produced a satisfactory effect with 50 mg/Kg dose plus 6mg/kg up to one hour after administration. Complications can occur regardless of the dose used.

## INTRODUCTION

Chloral hydrate (CH) is a well established sedative and hypnotic drug used in pediatric and dental patients because of its little depressive effect on the respiratory and cardiac systems^1^. Nonetheless, cardiac arrhythmias and sudden death in children, especially those with obstructive apnea are described in the literature^2^, mainly with the use of high doses, very likely because of the build up of serum trichloroethanol, an intermediate metabolite from the liver metabolism of the drug1. A possible carcinogenic action, already seen in guinea pigs has been described^3^, although not proven in humans^1^.

Among the less severe complications we have paradoxical hyperactivity, nausea, vomits and excessive sleepness^2^.

CH is one of the drugs of choice for the sedation of children submitted to Brainstem Auditory Evoked Potentials (BAEP), when any movement or muscle contraction can generate artifacts which impact on the analysis^4^.

A deep sleep with mean duration of one hour is quickly induced depending on the dose used, not leaving residual sleepiness after this period; nonetheless, there is no consensus in the literature as to the best dose, which can vary between 40 and 100mg/kg^2^^,^^4^, ^5^, ^6^, ^7^. Thus, we did this study in order to assess the efficacy of CH as a sedative agent used in the BAEP study in children and systematize its employment in daily practice.

## MATERIALS AND METHODS

This research project was approved by the Ethics in Research Committee of our institution (protocol 297/2008).

We did a cross-sectional prospective study with 41 children, without prior severe pulmonary or heart diseases, between 01 and 11 years; mean age of 3.75 years and standard deviation of ± 2.25 years, where 46.34% were females and 53.66% were males, consecutively referred to BAEP study under sedation.

We included only those children who failed in their tests under natural sleep or those with body weight below 30kg. CH was contraindicated in those children with heart diseases and/ or severe pulmonary diseases by their clinicians, and it was also contraindicated in those children using central nervous system drugs who were not approved for the procedure by their neurologist and/ or psychiatrist.

All the participants had been fasting for at least 6 hours and were recommended sleep deprivation in order to facilitate drug action.

The initial dose of CH at 10% was 50mg/kg with a booster of 6mg/kg given after 30 minutes in the cases when there was no sedation. The patients were monitored through a pulse oxymeter and the procedure room was equipped to care for respiratory complications (oropharyngeal cannula, valve-mask bag with oxygen reservoir, pediatric facial mask, pediatric endotracheal tube, laryngoscope with straight and curved blades, aspiration cannula, nasal catheter, material for venous access and medication to treat respiratory arrest).

The drug effectiveness was established by sleep induction up to one hour after administering the initial dose. Sleep was correlated with the 50mg or 56mg/kg doses, with the age ranges of < 2 years, from 2 to 5 years and > 5 years), in the following weight ranges <13Kg, from 13 to 19Kg and >19Kg) and with gender, using the pertaining statistical tests and statistical analyses (Levene test: test comparing two or more populations according with variance homogeneity, and chi-square test for samples with nominal variables in two or more categories) and a significance level of 5%.

## RESULTS

All the 41 children who participated in the study took 50mg/kg and 23 slept within 30 minutes. Two children (4.88%) had respiratory complications with reduction in the level of blood oxygen (<90%) still sleepy but awake. Desaturation was quickly reverted with an increase in oxygen supply through a facial mask and the protocol was suspended. 16 children took a booster dose of 6mg/ kg (total of 56mg/kg) and 13 slept for 30 minutes more. The 50mg/kg dose, followed by 6mg/kg had a statistically significant effect on the induction of sleep when compared to the 50mg/kg dose (p<0.05).

**Figure 1:**
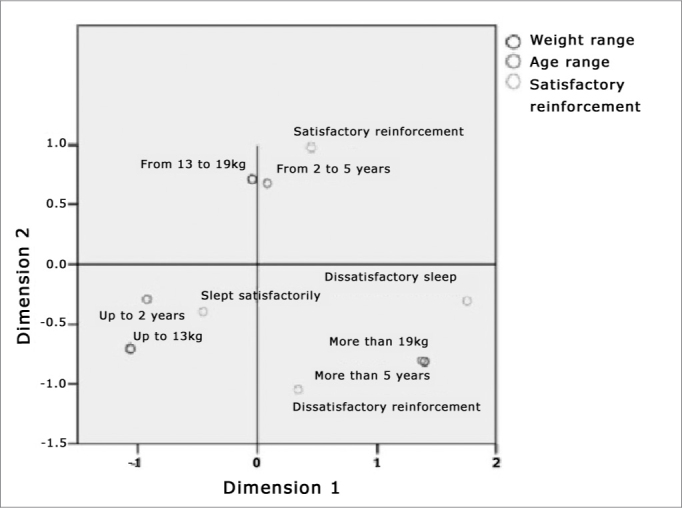
Perception map of the variables associated with age ranges, weight ranges and whether or not there was a booster dose with satisfactory effect or not.

There was no significant correlation between sleep induction and age (p=0.170), between sleep induction and weight (p= 0.51) and between sleep induction and gender (p= 0.142). Figure 1 depicts a perception map comparing the variables associated with the sample, in other words, age ranges, whether or not the 6mg/kg booster dose was used and if the effect was satisfactory (sleep induction) or not. The result indicated that there is no visible association between the respective variables when analyzed in a combined fashion since, in the graph, they do not form clusters among the different points which represent each variable mentioned.

## DISCUSSION

Although CH is one of the drugs of choice to se-date children to be submitted to ENT procedures, such as nasofibroscopy^5^ and BAEP, very little is discussed about its effective dose and its side effects. CH is metabolized into trichloroethanol which is responsible for the acute toxicity of this drug. The trichloroethanol has a half life of 9 to 40 hours, depending on patient age and the body development. Trichloroacetic acid is still detected 6 days after administration of one single dose of 50mg/kg^3^. Therefore, in the cases of sedation failure in the performance of the exam or the need to repeat the procedure, we do not recommend a new CH administration within less than a week of interval.

The major advantage of CH is a relatively fast sedation induction, especially when associated with sleep deprivation and, producing no residual sleepiness after this period, enabling each child to be quickly awakened only by increasing external stimuli, which is a different effect from that of other drugs that cause Central Nervous System depression^2^. Nonetheless, literature suggests that CH is chosen as the sedation drug of choice for procedures in children below 46 months of age only and that, after this age, another agent must be used in order to provide a safe sedation effect considering some toxic effects^2^.

We agree with the literature in keeping the maximum weight range in 30kg, however, because of the impossibility of using other CNS depressive drugs in our service, we have used CH in older children. Our results do not show statistical differences comparing the satisfactory effect, in other words, sleep induction, with the age of the patients for both doses of 50mg/kg and 56mg/kg. we also did not find significant results comparing sedation with gender and weight in our sample, but we reinforce that we did not administer CH at the dose of 50mg/kg and/or 56mg/kg for children heavier than 30kg.

Sleep induction with the total dose of 56mg/kg was statistically more significant when compared to the 50mg/ kg dose, despite the sample with a lower dose be larger and, even with the literature recommending doses between 40 and 100mg/kg^2^^,^^4^, ^5^, ^6^, ^7^, we believe that the 56mg/kg dose -within the proposed protocol of first giving 50mg/kg and if needed a 6mg/kg booster dose, yields good results, not requiring larger doses.

Two children (4.88% of the sample) had respiratory depression (<90% of saturation) with the 50 mg/kg dose reverted with oxygen given through a facial mask. This percentage is very close to the one reported in the literature (5.3%) even with larger samples (854 patients)^8^ and with doses considered safe (65± 13mg/kg). Most of these types of complications can be solved by increasing the flow of oxygen through the facial mask or nasal catheter, nonetheless, more invasive procedures may be needed. Because of these possible adverse effects, it is mandatory to have, in the exam room, the minimum necessary equipment to treat respiratory depression, besides a physician properly trained to care for such complication^8^.

In all cases of respiratory complications, it is highly recommended to suspend the procedure for a better control of the patient and that is why we did not continue with the protocol concerning the two children who had oxygen desaturation. It is also worth stressing that adverse respiratory events may happen even after the end of the procedure8 and with the child awake in the recovery room. Thus, we recommend monitoring the child for at least one hour after the end of sedation.

Complications such as paradoxical restlessness are described and present in about 2% of the patients who received CH, although we did not notice these adverse effects in this specific sample. Other complications associated with the use of CH such as hypotension, bradychardia and supraventricular tachycardia are described in the cases of sedation for invasive procedures such as cardiac catheterization and in children with high anesthetic risk (ASA III and IV)^8^.

## CONCLUSION

We can then conclude that the protocol proposed for the total dose of 56mg/kg (50 mg/kg followed by a booster dose of 6mg/kg) proved to be more effective in the sedation with only the 50 mg/kg dose and that this booster must be considered for those cases in which sedation did not happen within 30 minutes after 50mg/kg of CH administration. Adverse events such as respiratory complications may happen regardless of the dose used, requiring patient monitoring and proper equipment for emergency care.
